# Viscotrabeculotomy with anterior chamber irrigation versus Ahmed glaucoma valve implantation for silicone oil glaucoma in the pseudophakic eye

**DOI:** 10.5935/0004-2749.20230042

**Published:** 2023

**Authors:** Ahmed Samy Elwehidy, Nader Hussein Lotfy Bayoumi, Amr Abdelkader, Amani E Badawi

**Affiliations:** 1 Faculty of Medicine, Mansoura University, Mansoura, Egypt.; 2 Faculty of Medicine, Alexandria University, Alexandria, Egypt.

**Keywords:** Glaucoma drainage, implant, Glaucoma, Retinal detachment, Silicone oil, Trabeculectomy, Intravitreal Injection, Intraocular pressure, Postoperative complication, Ophthalmic solution, Dexamethasone, Ofloxacin, Implante para drenagem de glaucoma, Glaucoma, Descolamento retiniano, Óleo de silicone, Trabeculectomia, Injeção intravítrea, Pressão intraocular, Complicação pós-operatória, Solução oftálmica, Dexametasona, Ofloxacino

## Abstract

**Purpose:**

To compare viscotrabeculotomy with anterior chamber irrigation to Ahmed glaucoma valve implantation for secondary glaucoma following silicone oil removal.

**Methods:**

A prospective study was conducted on 43 vitrectomized pseudophakic eyes with persistent glaucoma after silicone oil removal. Patients were randomized to either viscotrabeculotomy with anterior chamber irrigation or Ahmed glaucoma valve implantation. All patients were examined on day 1, week 1, and months 1, 3, 6, 9, 12, 18, and 24 postoperatively. Postoperative complications were noted. Success was defined as an intraocular pressure between 6 and 20 mmHg and with an intraocular pressure reduction of >30% compared with the preoperative intraocular pressure.

**Results:**

There were 22 eyes in the viscotrabeculotomy with anterior chamber irrigation and 21 eyes in the Ahmed glaucoma valve implantation group. The mean preoperative and postoperative intraocular pressure in the viscotrabeculotomy with anterior chamber irrigation and Ahmed glaucoma valve implantation groups were 35.5 ± 2.6 mmHg and 35.5 ± 2.4 mmHg and 16.9 ± 0.7 mmHg and 17.9 ± 0.9 mmHg respectively (p˂0.0001). There was a statistically significant intraocular pressure reduction at all follow-up time points compared to preoperative values (p˂0.0001) in both groups. The unqualified success rate in the viscotrabeculotomy with anterior chamber irrigation and Ahmed glaucoma valve implantation groups were 72.73% and 61.9%, respectively. A minimal self-limited hyphema was the most common complication.

**Conclusions:**

Both viscotrabeculotomy with anterior chamber irrigation and Ahmed glaucoma valve implantation are effective in lowering the intraocular pressure in glaucoma after silicone oil removal with viscotrabeculotomy with anterior chamber irrigation providing greater reduction, higher success rates, and minimal complications.

## Abstract (pt)

**Objetivo:**

Comparar a viscotrabeculotomia com irrigação da câmara anterior com o implante de válvula de glaucoma de Ahmed para glaucoma secundário após remoção de óleo de silicone.

**Métodos:**

Foi realizado um estudo prospectivo de 43 olhos pseudofácicos vitrectomizados com glaucoma persistente após a remoção de óleo de silicone. Os pacientes foram randomizados para viscotrabeculotomia com irrigação da câmara anterior ou implante de válvula de Ahmed. Todos os pacientes foram examinados no primeiro dia, na primeira semana e 1, 3, 6, 9, 12, 18 e 24 meses após a cirurgia. Observaram-se complicações pós-operatórias. O sucesso foi definido como uma pressão intraocular entre 6 e 20 mmHg e uma redução da pressão intraocular >30% em comparação com a pressão intraocular pré-operatória.

**Resultados:**

Foram designados 22 olhos para o grupo da viscotrabeculotomia com irrigação da câmara anterior e 21 olhos para o grupo do implante de válvula de Ahmed. A pressão intraocular média pré-operatória foi de 35,5 ± 2,6 mmHg para o grupo da viscotrabeculotomia com irrigação da câmara anterior e pós- e de 35,5 ± 2,4 mmHg no grupo do implante de válvula de Ahmed. e Os valores pós-ope­ratórios foram de 16,9 ± 0,7 mmHg e 17,9 ± 0,9 mmHg para esses mesmos grupos, respectivamente (p<0,0001). Ambos os grupos tiveram uma redução estatisticamente significativa da pressão intraocular em relação aos valores pré-operatórios (p<0,0001) em todos os momentos do acompanhamento. A taxa de sucesso não qualificado nos grupos da viscotrabeculotomia com irrigação da câmara anterior e do implante de válvula de Ahmed foi de 72,73% e 61,9%, respectivamente. A complicação mais comum foi o hifema, autolimitado e mínimo.

**Conclusões:**

Tanto a viscotrabeculotomia com irrigação da câmara anterior quanto o implante de válvula de Ahmed são eficazes na redução da pressão intraocular no glaucoma após injeção de óleo de silicone, mas a viscotrabeculotomia com irrigação em câmara anterior proporcionou maior redução da pressão intraocular e maiores taxas de sucesso, com complicações mínimas.

## INTRODUCTION

Secondary glaucoma is a serious risk of intraocular silicone oil (SO) injection^(1)^; a procedure commonly resorted to surgical treatment of complex retinal detachment patterns with a variable incidence from 11% to 48% in different reports^(2,3)^, although high viscosity SO (5000 centistokes) results in less emulsification, and hence, fewer SO droplets in the anterior chamber (AC) with lower risk of glaucoma^(4)^, it does not preclude the occurrence of glaucoma in general. Theories for elevated intraocular pressure (IOP) with SO injection include clogging of the trabecular meshwork (TM) pores with emulsified SO droplets or SO-laden macrophages and cellular degeneration of TM cells due to the toxicity of SO^(5,6)^.

The risk of recurrent retinal detachment must be weighed against the benefit of lowering the IOP during early SO removal. Even AC irrigation to wash out any residual SO was reported to lower the IOP in such cases^(5)^. Persistently elevated IOP may be managed with topical IOP-lowering therapy, surgery, and/or laser. Filtering surgery (especially with antimetabolites augmentation) in SO-induced glaucoma is technically difficult and has a low success rate and high rate of complications^(7,8)^. Ahmed glaucoma valve (AGV) implantation offers a theoretical advantage of shifting filtration more posteriorly, providing a large filtration surface area and affording a valve control to aqueous humor exit^(9)^. AGV implantation for SO-induced glaucoma has been already reported^(10,11)^. Recently, there has been a rising interest in angle-based procedures (e.g., ab externo trabeculotomies) that target Schlemm’s canal (SC) as a more physiological approach in adult glaucoma with very promising results and a lower risk of complications compared to conventional glaucoma surgery^(12)^. Several reports have concluded that the use of viscoelastic materials during trabeculotomy (viscotrabeculotomy) may increase the success rate of the procedure by prevention of ocular decompression, postoperative hemorrhage, and AC shallowing^(13,14)^. The aim of this study was to compare the outcomes of viscotrabeculotomy with AC irrigation to AGV implantation for the management of secondary glaucoma following SO removal.

## METHODS

A prospective study was conducted on 43 eyes of 43 patients with persistent glaucoma after SO removal in vitrectomized pseudophakic eyes operated in Mansoura Ophthalmic Center of Mansoura University in Mansoura, Egypt, between March 2013 and December 2017. The study was approved by the ethics committee of the Faculty of Medicine of Mansoura University according to the Declaration of Helsinki, and all study participants gave written informed consent for participation in the study. The study eyes had undergone combined pars plana vitrectomy (PPV) with SO injection and phacoemulsification for rhegmatogenous retinal detachment and remained SO-filled for not more than 7 months. All studied eyes had developed persistently elevated IOP (>21 mmHg) for 6 weeks after SO removal with maximally tolerated topical IOP-lowering medications. Patients that were glaucomatous before PPV, patients without perception of light, patients with a postoperative follow-up of fewer than 24 months after glaucoma surgery, and eyes with previous scleral buckles were excluded from the study.

All study participants were subjected to a thorough ophthalmological examination, including measurement of the best corrected visual acuity (BCVA), slit-lamp examination, IOP measurement with Goldmann applanation tonometry (GAT; GAT, Haag-Streit, K¨oniz, Switzerland), dilated fundus examination, and gonioscopy for angle grading using Schaeffer’s grading system. Demographic data and the number of topical IOP-lowering medications were recorded. Patients were randomly assigned to receive either viscotrabeculotomy (VT) with AC irrigation (VTACI) or AGV implantation (AGVI).

All surgeries were performed by the same experienced surgeon (ASE). In VTACI, a fornix-based conjunctival flap was created followed by a half-thickness triangular (4.0-mm-sided equilateral) scleral flap dissection. A radial incision straddling the limbus was then created until SC was identified, followed by partial deroofing of the SC, the cut ends were cannulated, and a little amount of high viscosity sodium hyaluronate was injected (Healon GV; Pfizer, NY). At this step, a stab incision at the 12-o’clock position of the superior limbus was made, followed by insertion of an irrigation-aspiration cannula (Simcoe I/A cannula, dual shaft, front opening irrigation port, 23 gage, catalog product number G-16106 from Geuder Instruments, Heidelberg, Germany) into the AC to wash out and aspirate presumed SO droplets, especially those sequestered in the angle. Then, a suitable amount of high viscosity sodium hyaluronate was injected into the AC prior to an ab externo trabeculotomy, which was then performed on both sides of the deroofed part of the canal using a standard metal Harm’s trabeculotome (catalog product number G-15125 (right) and G-15130 (left) from Geuder Instruments, Heidelberg, Germany). The trabeculotomy was approximately one-third of the entire angle circumference. The viscoelastic was then irrigated out of the AC. Finally, the scleral and conjunctival flaps were tightly closed with nylon 10/0 sutures.

In AGVI, a 7/0 vicryl corneal traction suture at 12 o’clock was used to rotate the globe inferiorly. A superior fornix-based periotomy was extended to expose the superotemporal quadrant, followed by posterior dissection. After priming the valve (The AGV-FP7 model, New World Medical, Rancho Cucamonga, CA, USA) with a balanced salt solution using a 30-gage cannula, the AGV reservoir was gently tucked into the pocket with the tips of nontoothed forceps. The plate end was fixed 10 mm from the limbus with two 10/0 nylon sutures. The silicone tube of the valve was cut bevel up 1.5-2 mm anteriorly to the limbus. A scleral tunnel was fashioned 4 mm posteriorly to the limbus with a 23-G needle to enter AC parallel to the iris plane through which the tube was inserted into the AC. The tube was secured in place with a single nontight figure of eight (8/0 vicryl) scleral sutures, and then, the conjunctiva was closed with 8/0 vicryl sutures.

The postoperative treatment was the same in both groups and comprised topical dexamethasone and ofloxacin eye drops five times a day and cyclopentolate 3 times a day. The steroid and antibiotic eye drops were tapered gradually over 5 weeks while the cycloplegic eyedrops were discontinued at the end of the first postoperative week.

All patients were examined at 1 day, 1 week, 1 month, 3 months, 6 months, 9 months, 12 months, 18 months, and 24 months postoperatively. The examination included measurement of BCVA, slit-lamp examination, IOP measurement with GAT, and dilated fundus examination. Postoperative complications were noted.

Unqualified success was defined as IOP between 6 mmHg and 20 mmHg and with an IOP reduction of >30% compared with the preoperative IOP without any topical IOP-lowering medication or additional glaucoma surgery during the follow-up period. Qualified success was defined as IOP between 6 mmHg and 20 mmHg and with an IOP reduction of >30% compared with the preoperative IOP without any additional glaucoma surgery, with or without topical IOP-lowering medications during the follow-up. Failure was diagnosed when IOP was not controlled even with topical medications and if the patient needed additional surgical procedure(s) to lower the IOP, or when hypotony (i.e., IOP < 6 mmHg for more than 1 week after glaucoma surgery) persisted for >6 weeks. When an eye was diagnosed with treatment failure, it was excluded from the data analysis.

All statistical analyses were accomplished using IBMSPSS version 20. Assessment of the data normality was done using both the Histogram plot and Shapiro-Wilk’s test. Wilcoxon test was used to compare the preoperative and postoperative variables in each group. The comparison between the two groups was done using Mann-Whitney test for numerical variables and Chi-square test for categorical variables. Kaplan-Meier survival curve was plotted to estimate the mean survival time and probabilities of failure at different follow-up stages in both groups. For all tests, P value of less than 0.05 was considered significant.

## RESULTS

Forty-three eyes of 43 patients suffering persistent glaucoma after SO removal were included in the study. The eyes were randomized to receive VTACI (22 eyes) or AGVI (21 eyes). All patients had completed 24 months of follow-up. Eyes diagnosed with treatment failure (3 eyes and 5 eyes in the VTACI and AGVI groups, respectively) completed the 24 months follow-up and later, a part of the therapeutic services presented to the patients at the study setting; however, these eyes were excluded from data analysis. Table 1 shows the demographic and preoperative data of the patients in both groups. There was no statistically significant difference between both groups in all demographic and preoperative clinical parameters.

**Table 1:** Demographic and preoperative data of the 2 groups

	Demographic and preoperative data of the 2 groups		
	Group 1 (VTACI)	Group 2 (AGVFP7)	p
Patients (n, %)	22	21	
Male	15 (68.2%)	16 (76.2%)	0.736
Female	7 (31.8%)	5 (23.8%)	
**Eyes (n, %**)	22	21	1.000
Right	12 (54.5%)	12 (57.1%)	
Left	10 (45.5%)	9 (42.9%)	
**Age** (mean ± SD, range, median) in years	38.63 ± 12.06,24-59,36	39 ± 11.45,25-60,36	0.798
**RD types (n, %)**			
Myopic	17 (77.3%)	17 (81%)	1.000
Traumatic	5 (22.7%)	4 (19%)	
**Interval (1) (** mean ± SD, range, median) in months	4.82 ± 1.26, 3-7,5	4.81 ± 0.81,4-7,5	0.939
**Interval (2) (mean ± SD, range, median) in months**	2.41 ± 0.53,2-3,2	2.43 ± 0.58,2-4,2	0.978
**BCVA (mean ± SD, range, median) LogMar**	0.29 ± 0.1,0.1-0.5,0.3	0.28 ± 0.1,0.1-0.5,0.3	0.879
**IOP-Lowering drugs number (Mean ± SD, range, median)**	3.7 ± 0.5,3-4,4	3.7 ± 0.4,3-4,4	0.797

Mann-Whitney test and Chi-square test. P is significant at **<**.05.

RD= Retinal detachment, Interval (1): Time between vitrectomy and silicon oil removal, Interval (2): Time between silicon oil removal and the glaucoma operation; BCVA= Best corrected visual acuity.

The mean preoperative IOP was 35.5 ± 2.6 mmHg and 35.5 ± 2.4 mmHg in the VTACI and AGVI groups, respectively and this was significantly reduced to 16.9 ± 0.7 mmHg and 17.9 ± 0.9 mmHg respectively (p˂0.0001) at the end of 24 months. There was a statistically significant IOP reduction at all time points of the follow-up periods compared to the preoperative values (p˂0.0001) in both groups.

**Figure 1:**
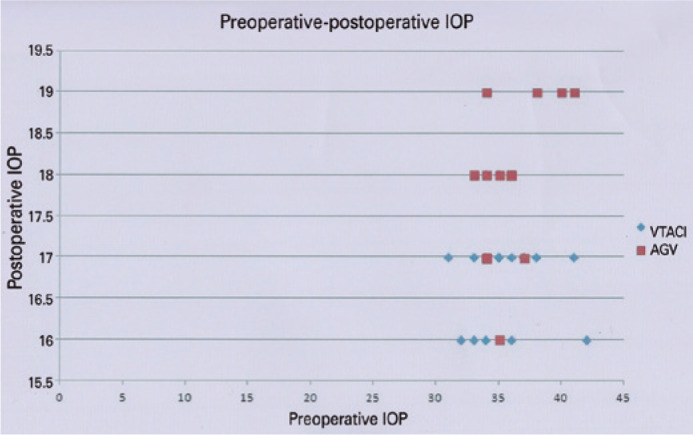
Scatter graph for the preoperative and 24-months postoperative IOP of all studied eyes in both groups.

The comparison between the two groups regarding the postoperative changes in mean IOP is displayed in table 2. Figure 1 presents the preoperative and 24 monthly follow-up IOP of eyes in both groups. There was a significant difference between the two groups in the mean IOP throughout the follow-up periods, starting at 1 week and continuing to the end of the follow-up period. At the end of 2 years, group 1 (VTACI) showed a statistically significant reduction of the mean postoperative IOP compared to group 2 (AGVFP7). The percentage of mean reduction in IOP pressure from baseline to the last follow-up was 52.2% (18.53 ± 0.66 mmHg) in group 1 and 50% (16.75 ± 0.52) in group 2 (p=0.002). There is no significant difference between the two groups concerning the BCVA and AGD at the end of 2 years. The baseline and follow-up IOP data of each patient in the AGV group is presented in supplemental table 1 (supplemental digital content) and that in the VTACI group is presented in supplemental table 2 (supplemental digital content).

**Table 2:** Comparison of IOP outcomes between the 2 groups at different follow-up time points

		Preoperative	1 Day	1 Week	1 month	3 months	6 months	9 months	12 months	18 months	24 months
**IOP (Mean± SD, range, median) mmHg, (number of eyes)**	**VTACI**	35.5 ± 2.6, 31-42,35, (22)	9.4 ± 1.1, 7-12,9.5, (22)	10.1 ± 0.79, 9-12,10, (22)	10.8 ± 0.9, 10-13,11, (22)	12.3 ± 1, 10-14,12, (22)	13.7 ± 1.2, 12-16,13.5, (22)	14.81 ± 1.4, 13-18,14, (22)	15.9 ± 2.4, 14-25,15, (22)	16.4 ± 2.3, 14-23,16, (21)	16.9 ± 0.7, 16-18,17, (19)
	**AGVI**	35.5±2.4, 30-41,36, (21)	8.5±1.7, 5-11,9, (21)	9±1.9, 5-12,9, (21)	11.5 ± 1.4, 9-14,12, (21)	13 ±1.3, 12-17,13, (21)	14.9±1.5, 12-19,14, (21)	16.2 ± 2.2, 14-24,15, (21)	17.4 ± 2.9, 15-26,16, (20)	17.8 ± 2.5, 16-25,17, (18)	17.9 ± 0.9, 16-19,18, (16)
**p**		0.844	0.072	0.012*	0.37*	0.001*	0.003*	0.006*	0.003*	0.001*	0.004*

Mann-Whitney test. P is significant at **˂**0.05

**Supplemental table 1:** Baseline and follow-up IOP data of each patient in the AGV Group

No.	Preop IOP	1 day	1 w	1 m	3 m	6 m	9 m	12 m	18 m	24 m
1	34	9	9	11	13	14	15	16	17	17
2	36	10	9	11	13	14	16	17	17	18
3	33	5	5	9	12	14	15	16	17	18
4	30	8	9	12	14	16	17	19	24	x
5	34	8	9	11	13	14	14	16	16	17
6	36	10	10	13	16	17	19	26	x	x
7	34	9	9	11	13	14	15	16	17	19
8	36	9	10	12	13	14	15	16	17	18
9	38	9	11	13	14	16	17	17	18	19
10	37	8	9	12	13	14	15	16	16	17
11	36	10	12	14	17	19	24	x	x	x
12	34	9	9	12	13	14	15	16	17	18
13	35	5	5	9	12	12	14	15	16	16
14	40	9	9	12	14	15	17	17	18	19
15	34	10	10	12	14	15	15	16	16	17
16	33	9	9	11	13	14	15	16	17	18
17	36	9	10	12	14	16	17	18	25	x
18	35	8	9	11	14	15	16	16	17	18
19	41	5	5	10	13	16	16	17	18	19
20	37	11	12	14	16	17	18	25	x	x
21	36	9	9	10	12	14	15	16	17	18

X= excluded from data analysis due to failure. Preop IOP= Preoperative intraocular pressure. 1 day - 24 m= postoperative follow-up time points starting from 1 day to 24 months.

**Supplemental table 2:** Baseline and follow-up IOP data of each patient in the VTACI Group

No.	Preop IOP	Preop. AGM	1 day	1 w	1 m	3 m	6 m	9 m	12 m	18 m	24 m
1	33	3	**9**	**10**	**11**	**12**	**13**	**14**	**14**	**14**	**16**
2	34	4	**10**	**11**	**12**	**14**	**16**	**17**	**18**	**23**	**x**
3	35	4	**8**	**9**	**10**	**11**	**12**	**14**	**14**	**16**	**18**
4	32	3	**10**	**10**	**11**	**12**	**12**	**13**	**14**	**15**	**16**
5	33	3	**9**	**9**	**10**	**11**	**13**	**14**	**15**	**15**	**17**
6	36	4	**10**	**10**	**11**	**13**	**14**	**15**	**16**	**17**	**18**
7	35	4	**9**	**10**	**10**	**13**	**14**	**16**	**17**	**17**	**18**
8	37	4	**10**	**10**	**11**	**12**	**14**	**15**	**16**	**16**	**17**
9	34	3	**7**	**9**	**10**	**12**	**13**	**14**	**14**	**15**	**17**
10	35	4	**10**	**11**	**11**	**12**	**13**	**14**	**15**	**16**	**17**
11	36	4	**9**	**10**	**10**	**12**	**13**	**14**	**15**	**16**	**17**
12	37	4	**10**	**10**	**11**	**13**	**14**	**15**	**16**	**16**	**17**
13	34	4	**9**	**10**	**10**	**10**	**13**	**14**	**15**	**15**	**16**
14	41	4	**10**	**10**	**10**	**12**	**13**	**13**	**14**	**16**	**17**
15	35	4	**12**	**12**	**13**	**14**	**16**	**18**	**19**	**23**	**x**
16	31	3	**8**	**9**	**11**	**12**	**14**	**15**	**16**	**16**	**17**
17	36	4	**9**	**10**	**10**	**12**	**13**	**14**	**15**	**15**	**16**
18	37	4	**11**	**11**	**12**	**14**	**16**	**18**	**25**	**x**	**x**
19	42	4	**8**	**10**	**11**	**12**	**13**	**14**	**15**	**15**	**16**
20	35	4	**10**	**10**	**11**	**13**	**14**	**14**	**14**	**16**	**17**
21	38	4	**9**	**9**	**10**	**12**	**14**	**15**	**16**	**16**	**17**
22	34	3	**10**	**11**	**12**	**13**	**15**	**16**	**16**	**17**	**18**

X= excluded from data analysis due to failure. Preop IOP= Preoperative intraocular pressure. 1 day - 24 m= postoperative follow-up time points starting from 1 day to 24 months.

The success rates (qualified and unqualified) in the VTACI and the AGVI groups were 86.3% and 76.2%, respectively. In the VTACI group, the unqualified success rate (success without the use of IOP-lowering medications) was 72.73% and qualified success (success with or without the use of IOP-lowering medications) was 86.37% at the end of 24 months (Table 3). Treatment failure was noted in 3 eyes (13.64%) that underwent trabeculectomy, in 2 eyes treated with mitomycin C (MMC), and in one eye with Ex PRESS (model P50) device placement. The mean time for the second surgical intervention was 16 ± 3.464 months. In the AGVI group, the unqualified success (success without the use of IOP-lowering medications) was 61.9% and qualified success (success with or without the use of IOP-lowering medications) was 76.2% (Table 3). Treatment failure was noted in 5 eyes (23.8%) that underwent AGV revision with bleb excision for bleb encapsulation (5 eyes) and then diode laser transscleral cyclophotocoagulation after valve explanation (2 eyes). The mean time for the second surgical intervention was 13.8 ± 4.024 months.

**Table 3:** Other clinical characteristics and success rates of both groups at 24 months

		Preoperative	24 months	p
**BCV** A (mean ± SD, range, median) LogMar	VTACI	0.29 ± 0.1,0.1-0.5,0.3	0.25 ± 0.09,0.1-0.5,0.3	0.498
	AGVI	0.28 ± 0.1,0.1-0.5,0.3	0.28 ± 0.09,0.1-0.5,0.3	
**IOP-lowering drugs** (Mean ± SD, range, median)	VTACI	3.7 ± 0.5,3-4,4	0.6 ± 1.1,0-3,0	0.387
	AGVI	3.7 ± 0.4,3-4,4	1.00 ± 1.3,0-3,0	
**IOP reduction** (percentage) Difference from preoperative IOP (Mean ± SD,) in mmHg	VTACI	-	52.2% 18.53 ± 0.66	0.002*
	AGVI	-	50% 17.75 ± 0.52	
**Unqualified Success**	VTACI	-	16 (72.7%)	0.526
	AGVI	-	13 (61.9%)	
**Qualified Success**	VTACI	-	19 (86.4%)	0.952
	AGVI	-	16 (76.2 %)	

Mann-Whitney test and Chi-square test. P is significant at **˂**0.05.

BCVA= Best corrected visual acuity, IOP= Intraocular pressure; Complete success= IOP between 6 mmHg and 20 mmHg and with an IOP reduction of >30% compared with the preoperative IOP with no need for IOP-lowering drugs; Qualified success= IOP between 6 mmHg and 20 mmHg and with an IOP reduction of >30% compared with the preoperative IOP with no need for IOP-lowering medications.

**Figure 2:**
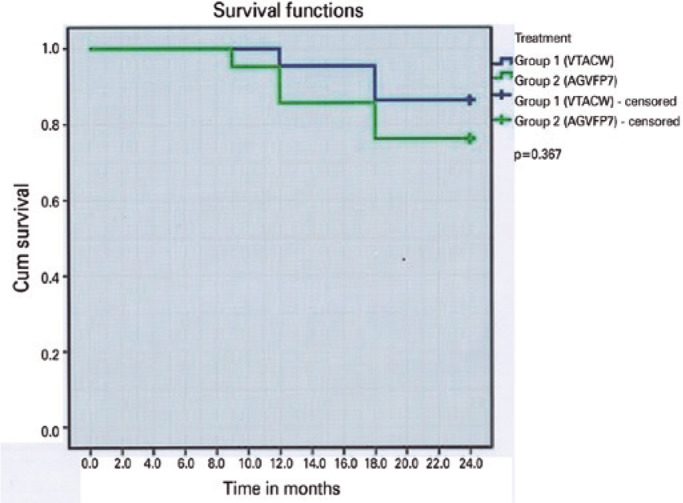
Kaplan-Meier survival analysis curve for the probability of postoperative success rate in both groups (survival was defined as the absence of failure).

Kaplan-Meier survival analysis curve for the probability of success rate in both groups is presented in figure 2. The mean survival time was 22.9 months in the VTACI group and 21.5 months in the AGVI group without significant difference (p=0.367).

Hyphema was the most common operative and postoperative complication in the VTACI group, being observed in 19 (86.36%) eyes. However, it resolved completely within 4-11 days postoperatively without the need for any surgical intervention. On the other hand, bleb encapsulation was the most common complication seen in the AGVI group (5 eyes, 22.72%) and it was the only cause of AGV failure. No sight-threatening complications were observed during the period of follow-up in both groups. Complications are listed in table 4.

**Table 4:** Postoperative complications and repeat interventions in both groups

	Postoperative complications and repeat interventions in both groups		
Complications (n, %)	VTACI (n=22)	AGVI (n=21)	p
Transient hyphema	19 (86.36%)	1 (4.76%)	**˂**.0001*
Localized Descemet’s membrane detachment	3 (13.63%)	0	0.233
Transient shallow anterior chamber	1(4.54%)	3 (14.28%)	0.345
Transient hypotony (<6 mmHg)	0	3 (14.28%)	0.104
Choroidal detachment	0	2 (9.52%)	0.233
Persistent IOP elevation (>20 mmHg)	3 (13.63%)	5 (22.72%)	0.457
Bleb encapsulation	-	5 (22.72%)	-
Recurrent RD	0	1 (4.76%)	0.488
Tube corneal touch	-	1 (4.76%)	-

Chi-square test. P is significant at ˂0.05

## DISCUSSION

The aim of this study was to compare two different techniques for the surgical lowering of persistently elevated IOP after SO removal in eyes operated for RD with PPV and SO injection. Both VTACI and AGVI reduced IOP by almost 50% with VTACI demonstrating a marginal advantage over AGVI. This IOP reduction was marked in the early postoperative period with a gradual rise over a 2-year follow-up period in both groups. Although the IOP at the final follow up was almost half the preoperative IOP. The study participants demonstrated a male predominance with almost two-thirds of cases in both groups. This is in accordance with other published reports^(10)^ and it parallels the reported higher incidence of RD in males^(15-17)^. The equal laterality distribution between right and left eyes is a recapitulation of the absence of laterality preference of RD and glaucoma, as already reported^(16)^. Noteworthy, the study population was relatively young. Lifelong medical treatment with its antecedent costs and complications is thus not practical, and a short-cut surgical procedure is justified. Additionally, this emphasizes the need for an efficient long-term procedure to control the IOP; hence, the procedures investigated in the current study. The presence of a wide age range of study participants demonstrates the fact that RD spans all age groups and no age is immune, in accordance with other reports^(16,17)^. A glimpse at the details of the RD of the studied eyes demonstrates that the majority belonged to the subset of myopic RD. This highlights the fact that myopia was a common etiology for RD, as already reported^(16,18)^. Moreover, myopia is a reported risk factor for the development of glaucoma^(19)^. Hence, it remains speculative that the studied eyes were already susceptible to glaucoma on account of pre-existing myopia. Another important issue is a relatively short duration between the PPV and SO removal, which is much shorter than the duration expected for SO to emulsify, a fact that may support the notion that these myopic eyes were natively susceptible to glaucoma, and the RD and RD surgery has just exposed this predisposition. The second duration that is also reportedly short is between SO removal and glaucoma surgery. A minimum of two months was left to allow for stabilization of the blood-aqueous barrier after SO removal and allow for maximal medical treatment to demonstrate its effect. Yet, it was kept to a minimum beyond that to protect the eye, and optic nerve specifically, from the detrimental effect of elevated IOP. This duration from SO removal to glaucoma surgery parallels other published reports^(10)^. Obviously, pre-existing myopia with its antecedent documented retinal changes^(20)^ and RD with its antecedent detrimental effect on vision even after repair^(21,22)^ provide a logical explanation for the relatively poor BCVA in the studied eyes. The fact that IOP-lowering therapy was used to its maximally tolerated dose before surgery reflects the poor response of that type of glaucoma to medical treatment, as already reported^(2,23)^.

Studying the IOP trends in the operated eyes reveals important insights. For both surgical techniques, IOP demonstrated an initial reduction followed by a gradual steady rise over time, although ending at 2 years at a level that was still less than the preoperative values. This trend is in agreement with other studies^(10)^. Whether longer follow-up would have allowed for IOP elevation to the preoperative values, or perhaps more, remains speculative. Scrutiny into each surgical technique demonstrates a marginal advantage of VT over AGV from 1 month postoperatively onwards. Indeed, the authors hypothesize that VT provides a more physiological drainage pathway for aqueous humor; hence, the slightly better IOP-lowering than AGV. Irrigation of SO from AC may be an additional contributing factor to the relative superiority of angle surgery. After all, clogging the TM with SO is a contributing factor to IOP elevation in SO-induced glaucoma. Hence, although VTACI does not obviously treat the entire circumference of the angle, yet the washout of SO may have improved the function of the remaining untreated part of the angle in AC. Moreover, the well-reported hypertensive phase of AGV^(24)^ has an onset at about 4-6 weeks after AGVI, which is the timing of the obvious rise of IOP in the AGV group in the current study. Additionally, AGV efficacy reduces with time, which is a well-reported property of AGV^(10)^. Though the concept of target IOP is instrumental in glaucoma treatment, yet the absence of BCVA reduction over the studied period provides at least a rough clue that IOP reduction was sufficient to preserve central vision, although no data on peripheral vision is presented. The reduction of IOP-lowering therapy at the end of follow-up is a clear advantage to the ocular surface and patients’ and/or health system finances. A confirmation of the advantage of VT over AGV is the higher success rate of the former over the latter. After all, the success of VT in managing different types of glaucoma has been recently emphasized^(14,25)^. And the decline of AGV success with time is also reported^(10)^. An important related issue is the time to treatment failure in those eyes where the treatment failed in either group. Treatment failures occurred earlier in the AGV implanted eyes than VT operated eyes, which is another advantage of VT over AGV. Finally, it is not surprising that hyphema was so common with VT given the reflux of blood through the valveless episcleral system into the SC to the AC with decompression of the latter, as already reported with VT in other types of glaucoma^(25)^.

This study has several limitations. There was no baseline information about the study eyes before the RD, regarding whether any of the study eyes had been originally glaucomatous prior to the occurrence of RD. This piece of information, although confirmed by, was not supported by medical records that were not available for the studied eyes. There was no presentation of the functional investigations for the studied eyes, namely, visual field data. However, it was the IOP that was the primary outcome of the study. The absence of central corneal thickness data, especially in such eyes with repeated intraocular procedures and AC wash, has implications for IOP measurement by GAT.

To conclude, to the best of the authors’ knowledge, this is the first study to compare VT to AGVI for elevated IOP after SO removal in eyes operated for RD with SO injection. Both VT and AGV are effective in lowering the IOP in such eyes with a clear advantage of VT over AGV, providing a greater reduction in IOP, higher success rates, and minimal complications.

### What was known

Silicone oil-induced glaucoma is refractory to medical treatment and conventional filtering surgery.

### What is new in this submission

Both viscotrabeculotomy with anterior chamber (AC) irrigation and Ahmed glaucoma valve (AGV) are effective to reduce intraocular pressure in silicone oil-induced glaucoma in pseudophakic eyes.

Viscotrabeculotomy with AC irrigation is slightly more effective than AGV in reducing the intraocular pressure in silicone oil-induced glaucoma in pseudophakic eyes.
